# Acute type a aortic intramural hematoma complicated with preoperative hemopericardium: early and late surgical outcome analyses

**DOI:** 10.1186/s13019-024-02616-y

**Published:** 2024-03-13

**Authors:** Chun-Yu Lin, Ming-Chang Kao, Hsin-Fu Lee, Meng-Yu Wu, Chi-Nan Tseng

**Affiliations:** 1 Department of Medicine, College of Medicine, Chang Gung University, Taoyuan City, Taiwan; 2 Department of Cardiothoracic and Vascular Surgery, New Taipei Municipal TuCheng Hospital, No.6, Sec.2, JinCheng Rd, TuCheng, New Taipei City, 236 Taiwan; 3 Department of Anesthesiology, New Taipei Municipal TuCheng Hospital, New Taipei City, Taiwan; 4 Department of Cardiology, New Taipei Municipal TuCheng Hospital, New Taipei City, Taiwan; 5 Department of Cardiothoracic and Vascular Surgery, Linkou Medical Center, Chang Gung Memorial Hospital, Taoyuan City, Taiwan

**Keywords:** Acute type a intramural hematoma, Acute type a aortic dissection, Hemopericardium, Bleeding, Coagulopathy, Delayed sternal closure, Mediastinal packing

## Abstract

**Background:**

Acute type A aortic intramural hematoma (ATAIMH) is a variant of acute type A aortic dissection (ATAAD), exhibiting an increased risk of hemopericardium and cardiac tamponade. It can be life-threatening without emergency treatment. However, comprehensive studies of the clinical features and surgical outcomes of preoperative hemopericardium in patients with ATAIMH remain scarce. This retrospective study aims to investigate the clinical features and early and late outcomes of patients who underwent aortic repair surgery for ATAIMH complicated with preoperative hemopericardium.

**Methods:**

We investigated 132 consecutive patients who underwent emergency ATAIMH repair at this institution between February 2007 and August 2020. These patients were dichotomized into the hemopericardium (*n* = 58; 43.9%) and non-hemopericardium groups (*n* = 74; 56.1%). We compared the clinical demographics, surgical information, postoperative complications, 5-year cumulative survival rates, and freedom from reoperation rates. Furthermore, multivariable logistic regression analysis was utilized to identify independent risk factors for patients who underwent re-exploration for bleeding.

**Results:**

In the hemopericardium group, 36.2% of patients presented with cardiac tamponade before surgery. Moreover, the hemopericardium group showed higher rates of preoperative shock and endotracheal intubation and was associated with an elevated incidence of intractable perioperative bleeding, necessitating delayed sternal closure for hemostasis. The hemopericardium group exhibited higher blood transfusion volumes and rates of re-exploration for bleeding following surgery. However, the 5-year survival (59.5% vs. 75.0%; *P* = 0.077) and freedom from reoperation rates (93.3% vs. 85.5%; *P* = 0.416) were comparable between both groups. Multivariable analysis revealed that hemopericardium, cardiopulmonary bypass time, and delayed sternal closure were the risk factors for bleeding re-exploration.

**Conclusions:**

The presence of hemopericardium in patients with ATAIMH is associated with an elevated incidence of cardiac tamponade and unstable preoperative hemodynamics, which could lead to perioperative bleeding tendencies and high complication rates. However, patients of ATAIMH complicated with hemopericardium undergoing aggressive surgical intervention exhibited long-term surgical outcomes comparable to those without hemopericardium.

## Introduction

Aortic intramural hematoma (IMH) accounts for 10–25% of all acute aortic syndromes [1]. In contrast to aortic dissection (AD), the intimal layer in IMH typically remains intact, with imaging studies usually not revealing a significant endothelial entry tear [2]. The primary pathogenesis linked to IMH is spontaneous bleeding from the vasa vasorum into the aortic media [2, 3]. In IMH, blood accumulates at the superficial region of the aorta adjacent to the adventitia, and patients exhibit elevated incidences of periaortic hematoma, hemorrhagic pericardial effusion, and aortic rupture in the mediastinum [4, 5]. Stanford acute type A aortic intramural hematoma (ATAIMH) is a life-threatening disease necessitating emergency treatment [6]. Established guidelines recommend approaching the management of this pathology in a manner similar to that of acute type A aortic dissection (ATAAD) [1, 7, 8]. Compared to patients with classic ATAAD, those with ATAIMH present with a higher prevalence of hemopericardium and cardiac tamponade [4, 5, 7], ranging from 61 to 69% and 33–45%, respectively, as reported in various international AD research registries [5, 9, 10]. However, comprehensive studies of the clinical features and surgical outcomes of preoperative hemopericardium in ATAIMH population remain scarce. In this study, we conducted a retrospective analysis utilizing the database from an individual aortic surgery center to compare the clinical demographics, surgical details, and early and late outcomes of patients who underwent aortic repair surgery for ATAIMH with or without preoperative hemopericardium.

## Materials and methods

### Patient enrollment and preoperative management

**Fig. 1:**
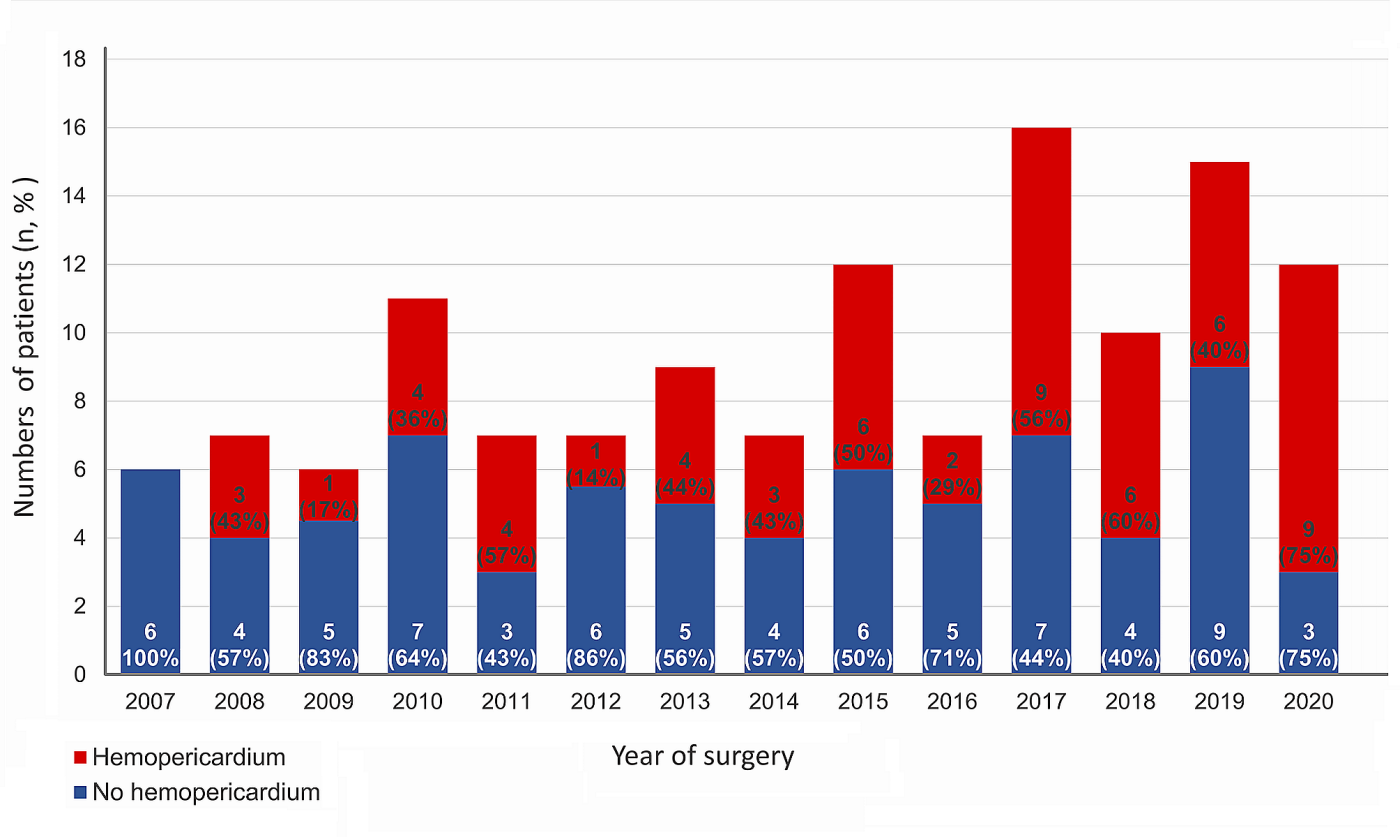
Annual distribution of patients for overall ATAIMH cohort, hemopericardium group, and non-hemopericardium group during the study period. ATAIMH, acute type A aortic intramural hematoma

The study protocol was approved by the Institutional Review Board of Chang Gung Memoriall Hospital (approval number 202301407B0). Between February 2007 and August 2020, 132 adult patients underwent emergency aortic repair surgery for ATAIMH at our institution. The diagnosis of IMH was defined as the presence of a circular or crescent-shaped thickening of > 5 mm of the aortic wall in the absence of detectable blood flow according to previous guidelines [1]. All patients were diagnosed using helical computed tomography in the emergency department and transferred to the operating room for emergency aortic repair. The 132 patients included in this study were divided into the hemopericardium (*n* = 58; 43.9%) and non-hemopericardium (*n* = 74; 56.1%) groups based on the presence of preoperative hemopericardium in computed tomography scan analyzed by experienced radiologists. Figure 1 shows the annual case distribution within the entire cohort and the hemopericardium and non-hemopericardium groups during the study period. The preoperative hemodynamics of the patients were stabilized by administering intravenous beta-blockers to maintain a systolic blood pressure of < 120 mmHg and heart rate of 60–70 bpm, according to established guidelines [1, 8]. Patients who presented with shock, defined as systolic blood pressure below 90 mmHg before surgery underwent immediate medical and surgical resuscitation management in accordance with the standardized protocols of our institution [11–13]. European system for cardiac operative risk evaluation score II was used for evaluate the surgical risk [14].

### Aortic repair procedures and postoperative treatment

The technical aspects of aortic repair procedures for ATAAD have been described in previous studies conducted at our institute [15, 16]. For patients with relatively stable preoperative hemodynamics, the primary approach involved double-artery cannulation, combining right axillary and femoral arterial access and integrating an antegrade cerebral perfusion strategy. Conversely, the preferred approach for patients with unstable preoperative conditions involved isolated femoral artery cannulation with retrograde cerebral perfusion. In all cases, a full sternotomy was performed. Cardiopulmonary bypass (CPB) with systemic hypothermia was initiated following cannulation of the right atrium or vena cava. In order to avoid further injury to the diseased aortic segment, we performed aortic cross-clamping with careful attention and minimal clamping force sufficient for blocking backflow from the distal ascending aorta (AsAo). Careful inspection of the aortic wall with removing residual thrombus was performed prior to undergo the anastomosis. The tubular AsAo, including the clamping site was routinely resected and replaced with a Dacron prosthetic graft. In general, the proximal anastomosis was performed first, followed by open distal anastomosis under deep hypothermic circulatory arrest (18–22˚C). During intraoperative examination, if substantial penetration of atherosclerotic ulcers or intimal tears was detected at the aortic root or arch, the diseased aortic segment was also replaced with a composite Valsava graft and a branched Dacron graft, respectively, as deemed feasible. All graft-aorta anastomoses were reinforced with Teflon strips and surgical sealants. During circulatory arrest, the femoral arterial flow was temporarily suspended and selective antegrade cerebral perfusion through the right axillary artery or retrograde cerebral perfusion through the superior vena cava was performed depending on the vascular access of cannulation. Concomitant frozen elephant trunk procedure with a thoracic aortic covered stent-graft was performed with direct antegrade delivery technique if the extent of IMH involved the descending thoracic aorta with suspicious entry tears and clinical manifestation associated with end-organ malperfusion.

Before terminating CPB and administering protamine, all graft-aorta anastomoses, cannulation sites, and fragile tissue surface were comprehensively examined and reinforced with pledgeted compression sutures if the active bleeders were identified. For patients complicated with intractable perioperative bleeding tendency, mediastinal packing with delayed sternal closure was performed according to the principles discussed in the previous study [17]. For postoperative treatment and monitoring, all patients were transferred to a specialized cardiovascular intensive care unit (ICU) following ATAIMH repair. The ventilator-weaning protocol was initiated at 12–24 h after surgery for patients who did not exhibit unstable hemodynamics, persistent arrhythmia, signs of organ malperfusion, or active bleeding. Renal replacement therapy was applied if acute renal failure developed after surgery, according to the Acute Kidney Injury Network criteria [18]. Re-exploration for bleeding was performed for patients exhibiting criteria of postoperative massive bleeding, including bleeding from chest tubes that exceeded 1.5 L during any 8 h period or massive transfusion with administration of more than 10 units of red cells within 24 h after surgery [19].

### Study data and endpoints

All data were obtained from the institutional AD database with electronic record. The primary endpoints for this retrospective study were early and late surgical outcomes, which were defined as in-hospital mortality rate and five-year cumulative survival/reoperation rates, respectively. Secondary endpoints were defined as postoperative complications and recovery, including blood transfusion volumes, re-exploration for bleeding, delirium, stroke, acute renal failure, organ malperfusion, infection, length of ICU and hospital stay.

### Statistical analyses

Statistical analyses were performed using SPSS for Windows (version 26.0; IBM Corp., Armonk, NY, USA). Continuous variables are presented as means ± standard deviation, while categorical variables are expressed as numbers (n) and percentages (%). To compare the intergroup disparities between the hemopericardium and non-hemopericardium groups, we utilized an independent t-test for continuous variables. Conversely, the chi-square test was employed for categorical variables. Multivariable logistic regression analysis was used to identify the independent risk factors associated with re-exploration for bleeding and in-hospital mortality after ATAIMH repair surgery. Preoperative and surgical variables respectively listed in Tables 1 and 2 were tested by univariable logistic regression analysis first. Variables with a *P* < 0.05 in the univariable logistic regression analysis were further analyzed via multivariable logistic regression analysis. The Kaplan–Meier method was employed to estimate the 5-year cumulative survival and freedom from aortic reoperation rates of the two groups, which were compared using the log-rank test. For all analyses, the statistical significance was set at *P* < 0.05.

**Table 1:** Preoperative characteristics

Parameters	Total	With hemopericardium	Without hemopericardium	*p*-value
	*n* = 132	*n* = 58	*n* = 74	
Clinical demographics				
Age (years)	61.4 ± 11.1	63.4 ± 11.9	59.9 ± 10.3	0.069
Sex (male, n,%)	79, 59.8	38, 65.5	41, 55.4	0.239
BMI (kg/m2)	26.8 ± 5.3	26.1 ± 5.5	27.3 ± 5.1	0.188
Hypertension (n,%)	99, 75	45, 77.6	54, 73	0.544
Diabetes mellitus (n,%)	9, 6.8	2, 3.4	7, 1.4	0.174
Creatinine (mg/dL)	1.5 ± 1.7	1.6 ± 1.9	1.4 ± 1.4	0.556
eGFR (mL/min/1.73 m^2^)	65.5 ± 27.9	62.1 ± 29.8	68.1 ± 26.2	0.220
Marfan syndrome (n,%)	3, 2.3	1, 1.7	2, 2.7	0.708
Preoperative condition				
Systolic blood pressure (mmHg)	96 ± 18.8	91.3 ± 21.3	99.7 ± 15.7	0.010
Systolic blood pressure < 90 mmHg (n,%)	34, 25.8	21, 36.2	13, 17.6	0.015
Cardiopulmonary resuscitation (n,%)	5, 3.8	3, 5.2	2, 2.7	0.461
Ventilator support (n,%)	10, 7.6	8, 13.8	2, 2.7	0.017
Repeat surgery (n,%)	1, 0.8	0	1, 1.4	0.374
EuroSCORE II (%)	9.9 ± 3.0	12.0 ± 2.6	8.3 ± 2.3	< 0.001
Time from ED to OR (h)	5.1 ± 2.5	4.7 ± 3.1	5.4 ± 1.9	0.142
Clinical presentation				
Chest/back pain (n,%)	107, 81.1	43, 74.1	64, 86.5	0.072
Cardiac tamponade (n,%)	21, 15.9	21, 36.2	0	< 0.001
Aortic regurgitation > moderate (n,%)	3, 2.3	2, 3.4	1, 1.4	0.422
Malperfusion^a^ (n,%)	10, 7.6	4, 6.9	6, 8.1	0.794
Cerebral infarction^b^ (n,%)	1, 0.8	0	1, 1.4	0.374
ATAIMH-related profiles				
DeBakey type II (n,%)	14, 10.6	8, 13.8	6, 8.1	0.292
AsAo diameter (mm)	45.4 ± 4.9	44.7 ± 3.5	45.9 ± 5.7	0.159
IMH thickness (mm)	11.2 ± 3.4	13.4 ± 3.1	9.5 ± 2.5	< 0.001
Hemopericardium thickness (mm)	NA	11.9 ± 6.7	NA	NA
ULP located at AsAo or arch (n,%)	17, 12.9	9, 15.5	8, 10.8	0.423
ULP located at descending aorta (n,%)	35, 26.5	12, 20.7	23, 31.1	0.179

^a^Limb ischemia in 7, cerebral infarction in 1, and paraplegia in 2 patients.

^b^Presence of impaired cerebral perfusion in a defined area associated with stenotic or occluded true lumen of supra-aortic arch branches, neck, and intracranial vessels.

AsAo, ascending aorta; ATAIMH, acute type A aortic intramural hematoma; ED, emergency department; eGFR, estimated glomerular filtration rate; EuroSCORE II, European system for cardiac operative risk evaluation score II; IMH, intramural hematoma; OR, operating room, ULP, ulcer-like projection.

**Table 2:** Surgical information

Parameters	Total	With hemopericardium	Without hemopericardium	*p*-value
	*n* = 132	*n* = 58	*n* = 74	
Femoral artery cannulation (n,%)	124, 93.9	57, 98.2	67, 90.5	0.065
Axillary artery cannulation (n,%)	109, 82.6	44, 75.9	65, 87.8	0.072
Aortic repair procedures				
Isolated AsAo replacement (n,%)	93, 70.5	38, 65.5	55, 74.3	0.271
Root replacement (n,%)	8, 6.1	4, 6.9	4, 5.4	0.722
Arch replacement (n,%)	32, 24.2	16, 27.6	16, 21.6	0.427
Partial arch (n,%)	23, 17.4	13, 22.4	10, 13.5	0.181
Total arch (n,%)	9, 6.8	3, 5.2	6, 8.1	0.507
Frozen elephant trunk (n,%)	6, 4.5	1, 1.7	5, 6.8	0.168
Resection of entry tear^a^ (n,%)	43, 32.6	17, 29.3	26, 35.1	0.479
Cardiopulmonary bypass time (min)	244.9 ± 63.2	244.6 ± 61.4	245.1 ± 69.2	0.196
Aortic clamping time (min)	161.1 ± 49.1	163.0 ± 48.1	159.6 ± 50.2	0.698
Circulatory arrest time (min)	49.7 ± 23.7	50.8 ± 24.9	48.9 ± 22.8	0.638
HTK cardioplegic solution (n,%)	91, 68.9	45, 77.6	46, 62.2	0.057
Hypothermia temperature (°C)	20.5 ± 2.5	21.0 ± 2.6	20.1 ± 2.4	0.063
Antegrade cerebral perfusion (n,%)	110, 83.3	46, 79.3	64, 86.5	0.272
Retrograde cerebral perfusion (n,%)	22, 16.7	12, 20.7	10, 13.5	0.272
Delayed sternal closure (n,%)	21, 15.9	14, 24.1	7, 9.5	0.022
Extracorporeal membrane oxygenation (n,%)	7, 5.3	4, 6.9	3, 4.1	0.470

^a^Ulcer-like projection found by preoperative imaging studies and subtle intimal tears with false lumen thrombosis identified by intraoperative inspection.

AsAo, ascending aorta; HTK, histidine–tryptophan–ketoglutarate.

## Results

### Patient demographics

Table 1 shows the preoperative demographics, which indicated no significant differences based on age, sex, or chronic comorbidities. Overall, the mean age was 61.4 ± 11.1 years, with males accounting for 59.8%. The hemopericardium group presented with more severe preoperative conditions, including lower systolic blood pressure, a higher incidence of shock and endotracheal intubation, and a higher EuroSCORE II estimated in-hospital mortality rate. Overall, 36.2% of the patients in the hemopericardium group exhibited cardiac tamponade before surgery. Chest or back pain was the most common symptom for patients with ATAIMH, accounting for > 70% in both groups. The average thickness of hemopericardium was 11.9 ± 6.7 mm. The hemopericardium group showed a greater thickness of IMH (13.4 ± 3.1 mm versus 9.5 ± 2.5 mm; *P* < 0.001). The extent of IMH, diameter of AsAo, location of ulcer-like projection did not reveal a significant difference between the two groups. In the non-hemopericardium group, 69 patients underwent emergent surgery, including 19 with persistent pain, 22 with IMH thickness > 11 mm, 6 with organ malperfusion, 14 with AsAo diameter > 50 mm, and 8 with ulcer-like projection located at AsAo or arch; five patients underwent delayed surgery (12–24 h after the initial diagnosis) for rapid progression of IMH thickness.

### Surgical information

Table 2 shows detailed information regarding the intraoperative variables. The vascular access of cannulation, extents of aortic repair procedure, rates of entry tear resection, CPB parameters, and cerebral perfusion strategies did not reveal significant differences between the two groups. The hemopericardium group exhibited a higher rate of intractable perioperative bleeding requiring mediastinal packing with delayed sternal closure for hemostasis (24.1% versus 9.5%; *P* = 0.022).

### Postoperative complications

Table 3 shows the postoperative mortality and morbidity rates. A higher in-hospital mortality rate is found in the hemopericardium group, although the statistical significance was not reached. The hemopericardium group had a higher mortality rate due to bleeding than the non-hemopericardium group. Furthermore, the hemopericardium group exhibited higher blood transfusion volumes for all types of components within 24 h after surgery, and a higher rate of re-exploration for bleeding (25.9% versus 9.5%; *P* = 0.012).

**Table 3:** Postoperative mortality and morbidity

Parameters	Total	With hemopericardium	Without hemopericardium	*p*-value
	*n* = 132	*n* = 58	*n* = 74	
In-hospital mortality (n,%)	14, 10.6	9, 15.5	5, 6.8	0.105
Bleeding (n,%)	4, 3.0	4, 6.9	0	0.022
Myocardial failure (n,%)	9, 6.8	5, 8.6	4, 5.4	0.467
Sepsis (n,%)	1, 0.8	0	1, 1.4	0.374
Transfusion within 24 h after surgery				
RBC^a^ (units)	10.4 ± 8.4	12.6 ± 8.0	8.7 ± 8.3	0.009
Plasma^b^ (units)	9.4 ± 7.7	11.7 ± 7.4	7.6 ± 7.5	0.020
Platelet (units)	21.9 ± 14.3	25.1 ± 14.2	19.5 ± 14.0	0.025
Re-exploration for bleeding (n,%)	22, 16.7	15, 25.9	7, 9.5	0.012
Delirium (n,%)	22, 16.7	9, 15.5	13, 17.6	0.754
Brain stroke (n,%)	13, 9.8	5, 8.6	8, 10.8	0.675
Infarction (n,%)	10, 7.6	4, 6.9	6, 8.1	0.794
Hemorrhage (n,%)	3, 2.3	1, 1.7	2, 2.7	0.708
Renal failure^c^ (n,%)	14, 10.6	6, 10.3	8, 10.8	0.931
Mesenteric ischemia (n,%)	4, 3.0	3, 5.2	1, 1.4	0.204
Limb ischemia (n,%)	4, 3.0	2, 3.4	2, 2.7	0.804
Pneumonia (n,%)	16, 12.1	8, 13.8	8, 10.8	0.602
Deep sternal wound infection (n,%)	3, 2.3	1, 1.7	2, 2.7	0.708
Ventilator support > 72 h (n,%)	43, 32.6	17, 29.3	26, 35.1	0.479
Tracheostomy (n,%)	7, 5.3	3, 5.2	4, 5.4	0.953
ICU stay (days)	9.1 ± 20.6	8.7 ± 11.8	10.4 ± 25.5	0.643
Hospital stay (days)	30.1 ± 39.3	30.7 ± 35.1	29.7 ± 42.6	0.884

^a^Red blood cell transfusion including the amount of whole blood and packed red cell concentrate.

^b^Plasma transfusion including the amount of fresh-frozen plasma and cryoprecipitate.

^c^Stage 3 acute kidney dysfunction according to the Acute Kidney Injury Network classification.

ICU, intensive care unit.

### Risk factors associated with re-exploration for bleeding

Table 4 shows the results of logistic regression analyses for patients at risks of undergoing re-exploration for intractable postoperative bleeding. The analysis revealed three significant risk factors: preoperative hemopericardium (odds ratio [OR], 3.21; 95% confidence interval [CI], 1.04–9.89; *P* = 0.042), CPB time (OR, 1.01; 95% CI, 1.00–1.02; *P* = 0.028), and perioperative bleeding tendency with delayed sternal closure procedure (OR, 3.73; 95% CI, 1.13–12.31; *P* = 0.031).

**Table 4:** Logistic regression analyses for re-exploration for bleeding

Parameters	β-coefficient	Standard error	Odds ratio, 95% CI	*p*-value
Univariable logistic regression				
Hemopericardium	1.206	0.498	3.34 (1.26–8.86)	0.015
Cardiopulmonary bypass time	0.012	0.004	1.01 (1.01–1.02)	0.001
Delayed sternal closure	2.015	0.533	7.50 (2.64–21.33)	0.001
Extracorporeal membrane oxygenation	2.070	0.805	7.93 (1.64–38.40)	0.010
Multivariable logistic regression				
Hemopericardium	1.167	0.574	3.21 (1.04–9.89)	0.042
Cardiopulmonary bypass time	0.009	0.004	1.01 (1.00–1.02)	0.028
Delayed sternal closure	1.316	0.609	3.73 (1.13–12.31)	0.031

CI, confidence interval.

### Risk factors associated with in-hospital mortality

Table 5 shows the results of logistic regression analyses for patients at risks of in-hospital mortality. The analysis revealed three significant risk factors: age (OR, 1.07; 95% CI, 1.00–1.15; *P* = 0.039), cardiopulmonary resuscitation (OR, 44.84; 95% CI, 4.48–449.36; *P* = 0.001), and malperfusion (OR, 6.46; 95% CI, 1.39–30.10; *P* = 0.017).

**Table 5:** Logistic regression analyses for in-hospital mortality

Parameters	β-coefficient	Standard error	Odds ratio, 95% CI	*p*-value
Univariable logistic regression				
Age	0.045	0.027	1.06(1.00–1.11)	0.047
Cardiopulmonary resuscitation	2.761	0.966	15.82(2.38–105.02)	0.004
Malperfusion	1.365	0.581	3.92(1.25–12.24)	0.019
Total arch replacement	1.627	0.774	5.10(1.12–23.23)	0.036
Cardiopulmonary bypass time	0.009	0.004	1.01(1.001–1.02)	0.029
Delayed sternal closure	1.639	0.607	5.15(1.57–16.91)	0.007
Extracorporeal membrane oxygenation	2.051	0.826	7.77(1.54–39.27)	0.013
Multivariable logistic regression				
Age	0.071	0.035	1.07(1.00–1.15)	0.039
Cardiopulmonary resuscitation	3.803	1.176	44.84(4.48–449.36)	0.001
Malperfusion	1.866	0.785	6.46(1.39–30.10)	0.017

CI, confidence interval.

### Cumulative 5-year survival and freedom from reoperation rates

**Fig. 2:**
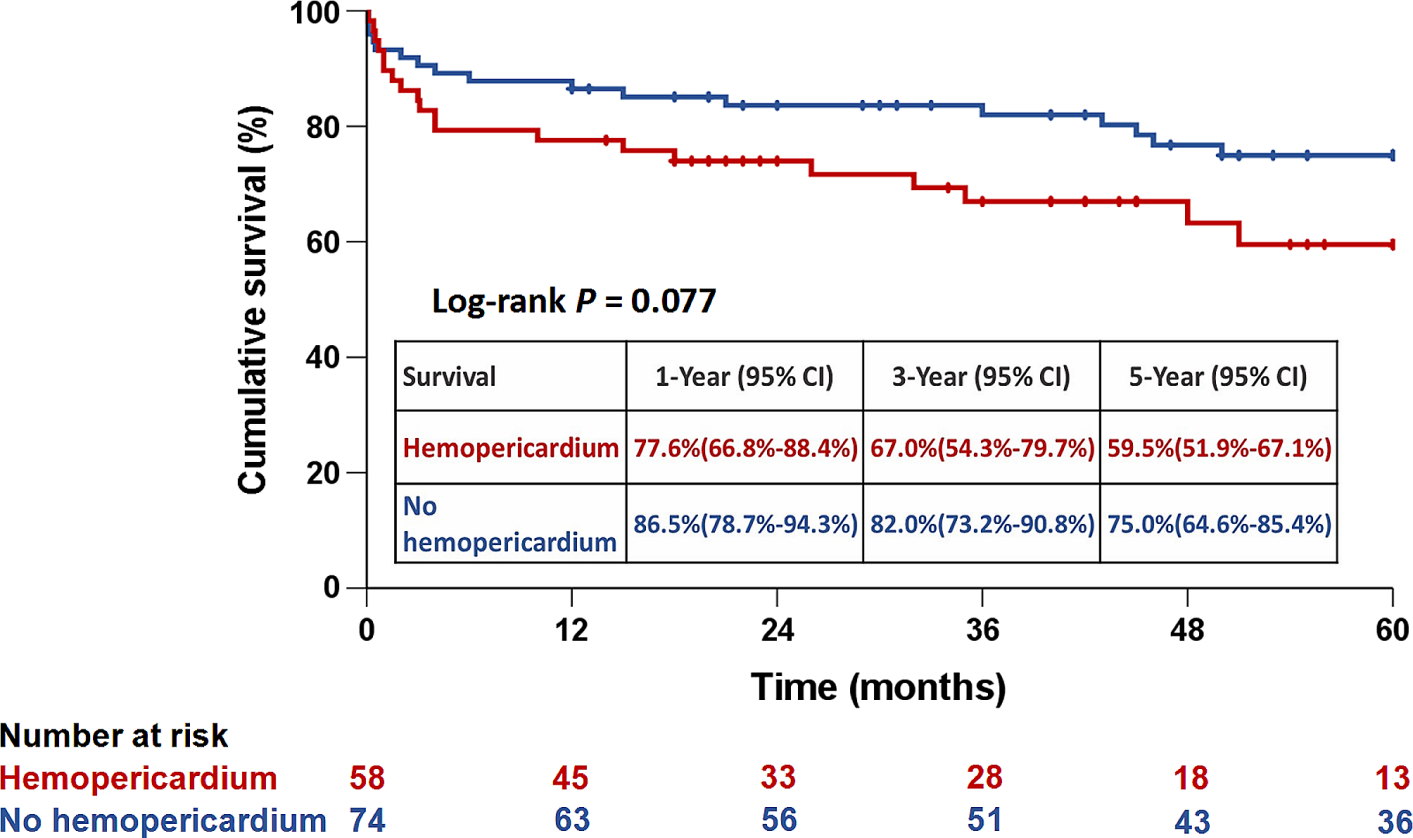
Five-year cumulative survival rates stratified by hemopericardium and non-hemopericardium groups

**Fig. 3:**
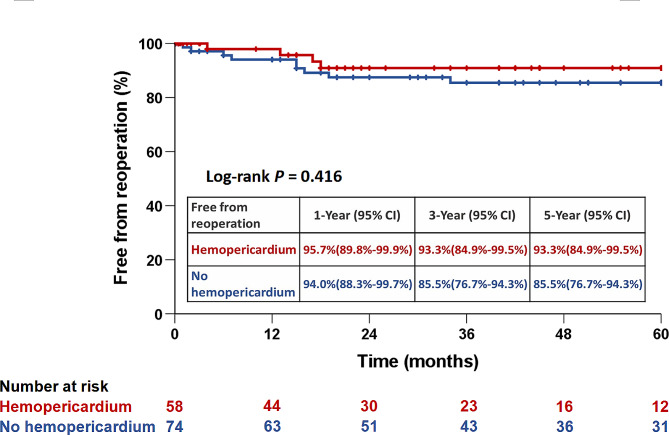
Five-year freedom from aortic reoperation rates stratified by hemopericardium and non-hemopericardium groups

The average follow-up duration was 4.7 ± 3.5 years (median, 4.1; range, 0.1–14.3 years). As illustrated in Figs. 2 and 3, respectively, the 5-year cumulative survival rates (59.5% versus 75.0%; *P* = 0.077) and freedom from aortic reoperation rates (93.3% versus 85.5%; *P* = 0.416) showed no significant difference between the hemopericardium and non-hemopericardium groups.

## Discussion

IMH is typically recognized as a circumferentially contained hemorrhage of the aortic wall without certain imaging evidence of an identifiable entry point. ATAIMH is a life-threatening cardiovascular emergency and associated with an increased risk of pericardial hemorrhage and cardiac tamponade compared to classic ATAAD [5, 6, 20]. However, the clinical features and surgical outcomes of pure ATAIMH population complicated with hemopericardium was scarcely reported in previous literatures. In this single-center cohort study, we investigated 132 consecutive patients (58 with hemopericardium and 74 without) who underwent emergency aortic repair for ATAIMH within the study period. This study yielded several principal findings. First, the incidence of preoperative hemoepricardium (43.9%) was considerable in the patients with ATAIMH, and was associated with a high risk of unstable preoperative conditions and cardiac tamponade. Second, with aggressive and standardized surgical treatment, patients with ATAIMH complicated with hemopericardium exhibited acceptable early and late outcomes as compared to those without hemopericardium. Third, patients who presented with preoperative hemopericardium were associated with a higher risk of intractable perioperative bleeding and re-exploration for bleeding. These patients should undergo intensive surveillance and adhere to strict criteria for early detection and treatment of postopertative bleeding before subsequent complications develop.

IMH is a challenging acute aortic syndrome with a clinical presentation similar to classic AD. It is associated with significant morbidity, mortality, and variable clinical behavior [7]. However, IMH differs from classic AD in pathophysiology. Instead of significant intimal rupture or dissection flap, it typically results from spontaneously contained hemorrhage within the media layer of the aortic wall [20]. These pathological processes result in circumferentially oriented blood-containing space that does not communicate with the true aortic lumen [4]. The persistent pressurized intramedial bleeding within the aorta could result in elastic microstructure injuries of the adventitia and aortic wall weakening following severe bleeding or even frank rupture of AsAo into the pericardial space. Rapidly progressive hemorrhagic pericardial effusion can lead to cardiac tamponade and subsequent hemodynamic instability. Previous studies have shown that ATAIMH is associated with a higher incidence of pericardial hemorrhage and cardiac tamponade than the classic ATAAD [5, 20]. The reported incidences of hemopericardium and cardiac tamponade associated with ATAIMH are substantial, accounting for > 60% and 30% of the patients, respectively [5, 9, 10, 21]. In contrast, only 18–28% of patients with classic ATAAD present with cardiac tamponade [9, 10]. A similar outcome was observed in the present study. Overall, 43.9% of patients were diagnosed with ATAIMH complicated by preoperative hemopericardium, and 36.2% of these cases presented with cardiac tamponade. Previous studies conducted at our institute, which investigated the general ATAAD population, showed that preoperative hemopericardium and cardiac tamponade incidence rates were approximately 33% and 12%, respectively [17, 22]. Current guidelines generally recommend early surgery for patients with ATAIMH owing to its potentially unpredictable pathological progress [1, 8], even a medical management might be considered reasonable in highly selected patients. At our institute, an aggressive surgical treatment strategy was adopted for this patient group. Surgical management is considered as a priority treatment option, except in cases where imaging examination indicates minimal thickness of the IMH with highly stable hemodynamics and the absence of any progressive clinical symptoms. Patients receiving medical treatment were closely observed in the ICU and underwent periodic follow-up image survey within 24 h after the initial diagnosis. Surgical intervention was promptly implemented for patients with disease progression.

The concept of consumption coagulopathy in patients with ruptured or dissecting aortic aneurysm was introduced in the 1960 and 1970 s [23, 24], and its associated intractable bleeding tendency has been acknowledged as a major complication in treating acute aortic syndromes [25]. We suggest that ATAIMH or ATAAD complicated by hemorrhagic pericardial effusion could be categorized as ruptured aortic aneurysms under an extended definition. Dysfunction of coagulation system is commonly observed in patients with acute AD, especially in those undergoing emergency aortic repair surgeries. The pathology and correlated mechanisms could be complex and multifactorial, involving injury to the aortic intimal structure, leading to the entry of blood into the non-endothelialized false lumen. When blood is exposed to subendothelial tissue factor, collagen, and the adventitial layer of the aortic wall, it results in consumption coagulopathy, manifesting as a reduction in clotting factors, platelet dysfunction, and disseminated intravascular coagulation [23–25]. Furthermore, complex aortic repair procedures, systemic thrombolysis associated with prolonged CPB duration, tissue trauma with excessive bleeding, and extensive blood transfusions also compromise the coagulation system. Hemopericardium caused by bleeding or frank rupture of the AsAo would further exacerbate the bleeding cascade. In our study, we identified preoperative hemopericardium as a risk factor associated with the need for postoperative re-exploration due to bleeding. Furthermore, we observed that postoperative blood transfusion volumes, encompassing red blood cells, plasma, and platelets, were generally higher in the hemopericardium group than in the non-hemopericardium group. This could address a more severe bleeding tendency among these patients. Despite advancements in management algorithms, surgical strategies, and CPB techniques, reoperation for postoperative bleeding remains as a serious complication. Previous studies from various international AD data registries have reported bleeding reoperation rates ranging from 9 to 20% in the general ATAAD population [26–28]. In the present study, over 25% of patients in the hemopericardium group underwent re-exploration for bleeding after surgery. This incidence was higher than that in previous studies from this institute [16, 22]. Owing to their heightened risk of intractable perioperative bleeding, we suggest that these patients should be monitored rigorously and adhere to strict protocols for the early diagnosis and treatment of postoperative bleeding before resulting in subsequent complications.

A trend of higher in-hospital mortality rate is found in the hemopericardium group (15.5% versus 6.8%). As reported by Chien et al., preoperative shock (systolic blood pressure < 90 mmHg) was an independent predictor of in-hospital mortality for patients undergoing ATAAD repair [11]. In the present study, the hemopericardium group showed lower systolic blood pressure (91.3 ± 21.3 mmHg versus 99.7 ± 15.7 mmHg; *P* = 0.010) and a higher incidence of shock (36.2% versus 17.6%; *P* = 0.015) compared to the non-hemopericardium group. Therefore, these patients were expected to be at higher risk for in-hospital mortality.

### Limitations

This study has some limitations. First, since this is a retrospective and non-randomized controlled study, a potential bias may have existed and may have affected the homogeneity between the hemopericardium and non-hemopericardium groups. Furthermore, the relatively small sample size may also affect the power of statistical analyses and strength of data interpretation. Second, the treatment protocols for ATAAD and ATAIMH were based on institutional consensus and established guidelines. However, the ultimate decision making was left to the discretion of the operating surgeon with full consideration of each individual patient’s clinical condition. Therefore, a proactive approach to postoperative re-exploration may be implemented in patients with potential bleeding risks, including those with prior cardiac surgery, fragile aortic tissue, preoperative aortic rupture, and significant coagulopathy detected through laboratory tests. Furthermore, this retrospective cohort study spanned approximately 14 years. Hence, changes and advancements in CPB technology, myocardial protection, cerebral perfusion strategies, and ICU care protocols may have occurred. Finally, despite the substantial early and late findings of this study, an extended follow-up study with more included patients should be conducted in the future to analyze the long-term outcomes of patients undergoing surgical treatment for ATAIMH complicated with hemopericardium.

## Conclusions

ATAIMH complicated with hemopericardium is associated with an increased risk of cardiac tamponade, unstable preoperative hemodynamics, perioperative bleeding tendency, and high complication rates. However, the late outcomes, including survival and aortic reoperation rates during the 5-year follow-up, ware comparable between patients with and without preoperative hemopericardium.

## Data Availability

The datasets generated and analyzed in the current study cannot be made publicly available due to ethical and legal reasons. The Institutional Review Board of Chang Gung Medical Foundation must review all request for public data sharing to protect the patients’ privacy. Requests for data can be sent to the Institutional Review Board of Chang Gung Medical Foundation at irb1@cgmh.org.tw.

## Abbreviations

AD: Aortic dissection
AsAo: Ascending aorta
ATAAD: Acute type A aortic dissection
ATAIMH: Acute type A aortic intramural hematoma
EuroSCORE II: European system for cardiac operative risk evaluation score II
CI: Confidence interval
CPB: Cardiopulmonary bypass
ICU: Intensive care unit
IMH: Aortic intramural hematoma
OR: Odds ratio
ULP: Ulcer-like projection
